# Analysis of Operational Characteristics of AlGaN/GaN High-Electron-Mobility Transistor with Various Slant-Gate-Based Structures: A Simulation Study

**DOI:** 10.3390/mi13111957

**Published:** 2022-11-11

**Authors:** Jun-Ho Lee, Jun-Hyeok Choi, Woo-Seok Kang, Dohyung Kim, Byoung-Gue Min, Dong Min Kang, Jung Han Choi, Hyun-Seok Kim

**Affiliations:** 1 Division of Electronics and Electrical Engineering, Dongguk University-Seoul, Seoul 04620, Korea; 2 Electronics and Telecommunications Research Institute, Daejeon 34129, Korea; 3 Photonic Components Department, Fraunhofer Heinrich-Hertz Institute HHI, 10587 Berlin, Germany

**Keywords:** GaN, high-electron-mobility transistor, slant-gate, field-plate, breakdown voltage

## Abstract

This study investigates the operational characteristics of AlGaN/GaN high-electron-mobility transistors (HEMTs) by applying a slant-gate structure and drain-side extended field-plate (FP) for improved breakdown voltage. Prior to the analysis of slant-gate-based HEMT, simulation parameters were extracted from the measured data of fabricated basic T-gate HEMTs to secure the reliability of the results. We suggest three different types of slant-gate structures that connect the basic T-gate electrode boundary to the 1st and 2nd SiN passivation layers obliquely. To consider both the breakdown voltage and frequency characteristics, the DC and RF characteristics of various slant-gate structures including the self-heating effect were analyzed by TCAD simulation. We then applied a drain-side extended FP to further increase the breakdown voltage. The maximum breakdown voltage was achieved at the FP length of 0.4 μm. Finally, we conclude that the slant-gate structures can improve breakdown voltage by up to 66% without compromising the frequency characteristics of the HEMT. When the drain-side FP is applied to a slant-gate structure, the breakdown voltage is further improved by up to 108%, but the frequency characteristics deteriorate. Therefore, AlGaN/GaN HEMTs with an optimized slant-gate-based structure can ultimately be a promising candidate for high-power and high-frequency applications.

## 1. Introduction

AlGaN/GaN high-electron-mobility transistors (HEMTs) are widely used as power devices due to their high off-state breakdown voltage ($V_{\mathrm{BD}}$) that is a result of their remarkable material and electronic properties, such as wide energy bandgap (3.3 eV) and high critical electric field (3.39 MV/cm) [1,2,3]. These characteristics make GaN more practicable for high-voltage and high-temperature applications than silicon or gallium arsenide [4]. Additionally, HEMTs based on the AlGaN/GaN heterostructure show superb performances owing to the two-dimensional electron gas (2-DEG) via tensile and compressive stresses in the channel region that exhibits high electron mobility and high electron density, which have important roles in the output current and power amplification. Nevertheless, to fully cater to the market requirements, GaN-based HEMTs need to be capable of both high voltage and high-frequency applications [5,6,7]. Therefore, we designed slant-gate structures with high $V_{\mathrm{BD}}$ and high cut-off frequency ($f_{T}$) simultaneously, which were evaluated by Johnson’s figure of merit (JFOM) (=$f_{T}\times V_{\mathrm{BD}}$) [8,9,10].

Generally, the field-plate (FP) technology has the effect of increasing the $V_{\mathrm{BD}}$ by providing an extra metal edge, which leads to the redistribution and reduction in the electric fields concentrated at the drain-side gate electrode edge over the 2-DEG channel [11,12,13]. However, FP structures create additional parasitic capacitances, which can degrade RF characteristics in high-frequency operation and can also reduce power efficiency over the operating frequency range [14,15,16]. But the optimum slant-gate structure in this study can improve $V_{\mathrm{BD}}$ without degrading the frequency characteristics of the HEMT. Also, we confirmed that the $V_{\mathrm{BD}}$ of the slant gate structure is greatly improved with only a slight geometrical change in the existing basic T-gate electrode structure, with same gate length ($L_{\mathrm{gate}}$) of 0.18 μm and same epitaxial layer configurations.

The optimal gate structure was determined by analyzing the trade-off between $V_{\mathrm{BD}}$ and frequency characteristics of the various slant-gate-based structures. First, the simulated data were matched with measured drain current-gate voltage ($I_{\mathrm{DS}}$-$V_{\mathrm{GS}}$) transfer characteristics and frequency characteristics data of fabricated basic T-gate AlGaN/GaN HEMTs for verification. Afterward, we applied three different slant-gate structures to improve not only the $V_{\mathrm{BD}}$ while maintaining or improving other DC characteristics but also the RF performances. To further increase the $V_{\mathrm{BD}}$, we then applied a drain-side extended FP to the most effective of the three slant-gate structures. The $V_{\mathrm{BD}}$ was simulated while increasing the drain-side FP length up to 1.0 μm to determine the optimum FP length that results in the highest $V_{\mathrm{BD}}$. The AlGaN/GaN HEMTs with slant-gate-based structure which has high $V_{\mathrm{BD}}$ and high $f_{T}$ are expected to be used not only in 5G wireless network applications such as high-power amplifiers, but also in military systems such as radar transmitters.

## 2. Materials and Methods

**Figure 1:**
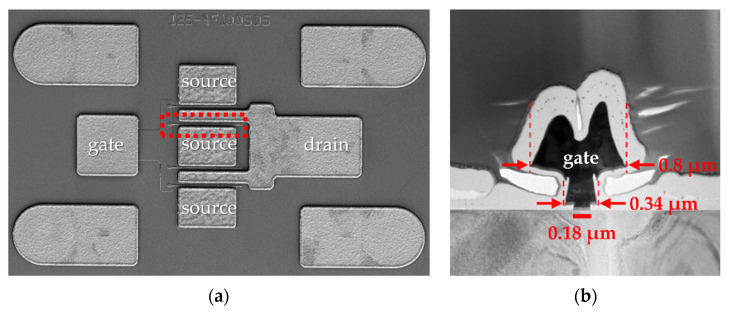
Scanning electron microscope (SEM) images of the fabricated AlGaN/GaN high-electron-mobility transistor (HEMT) structure: (**a**) top view of a four-finger transistor device and red dashed frame shows the representative one-finger gate electrode; (**b**) cross-sectional view of a basic T-gate electrode.

In order to verify the simulation parameters, HEMT with basic T-gate structure was fabricated. Figure 1 shows scanning electron microscope (SEM) images of a fabricated AlGaN/GaN HEMT. Figure 1a shows a top view of a four-finger transistor device consisting of the gate, source, and drain contact pads with a unit gate width of 100 μm and source-to-drain distance ($L_{\mathrm{Source}–\mathrm{Drain}}$) of 5 μm. Figure 1b is a magnified cross-sectional view of the red dotted region in Figure 1a, showing the conventional T-gate structure. As shown in Figure 1b, a T-shaped gate was formed by combining a narrow gate-foot length ($L_{\mathrm{Gate}–\mathrm{Foot}}$) of 0.18 μm, a gate-middle length ($L_{\mathrm{Gate}–\mathrm{Middle}}$) of 0.34 μm, and a gate-head length ($L_{\mathrm{Gate}–\mathrm{Head}}$) of 0.8 μm.

**Figure 2:**
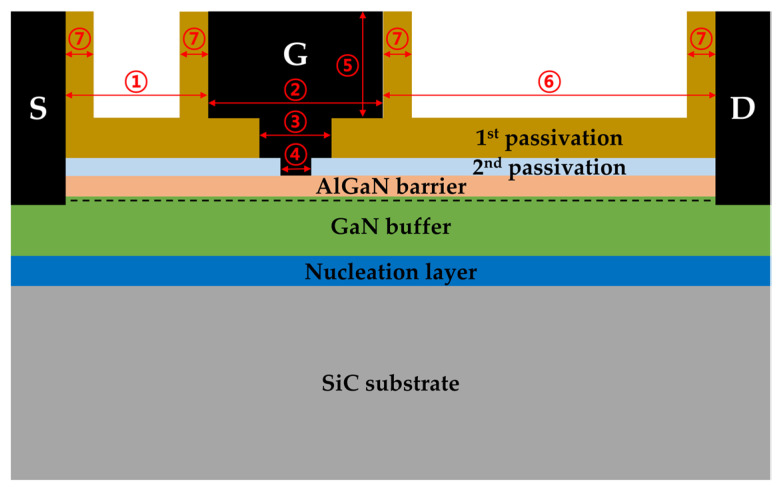
A cross-sectional schematic of the basic T-gate AlGaN/GaN HEMT used in the modeling. The S, D, and G stand for source, drain, and gate electrode, respectively, and the numbers are explained in the Table 1.

The unit device structure of a one-finger transistor was used in the modeling, and a cross-sectional schematic of a basic T-gate structure is shown in Figure 2. And Table 1 provides detailed geometrical parameter information of the T-gate structure used in the simulation. The AlGaN/GaN heterostructure HEMTs were grown on a 4-inch SiC substrate using metal-organic chemical vapor deposition. The epitaxial layers were consisted of a nucleation layer, a 2 μm thick Fe-doped GaN buffer layer, and a 25 nm thick Al_0_._25_Ga_0_._75_N barrier layer. Ti/Al/Ni/Au ohmic metallization was formed by rapid thermal annealing at 900 °C for 30 s, and device isolation was carried out by P^+^ ion implantation. Then, a 50-nm-thick SiN layer was deposited using plasma-enhanced chemical vapor deposition (PECVD). The first metal interconnections with the ohmic contacts were formed by the evaporation of Ti and Au metal after etching the SiN layer. The T-shaped gate process was performed by using two-step electron-beam lithography. First, the $L_{\mathrm{Gate}–\mathrm{Foot}}$ of 0.18 μm was formed by electron-beam exposure in poly methyl methacrylate (PMMA) resist and the SiN layer underneath the gate pattern was etched by reactive ion etching (RIE). Then, a T-shaped gate pattern with the $L_{\mathrm{Gate}–\mathrm{Middle}}$ of 0.34 μm was directly written by additional electron-beam exposure after coating with PMMA/Co-polymer/PMMA triple layers. The gate recess was formed using inductively coupled plasma (ICP) etching with BCl_3_/Cl_2_ gas and the two-step procedure to form the recessed gate was optimized by using ICP dry etching with a BCl_3_/Cl_2_ gas mixture and a wet cleaning process consisting of oxygen plasma treatment and diluted-HCl etching. For gate metallization, an Au/Ni metal stack with the respective thickness of 500/30 nm was deposited by electron-beam evaporation and lifted off. A SiN PECVD film was deposited for device passivation and etched using RIE for the source and drain pad contacts. A more detailed description of the process can be found in the previous paper [17].

**Figure 3:**
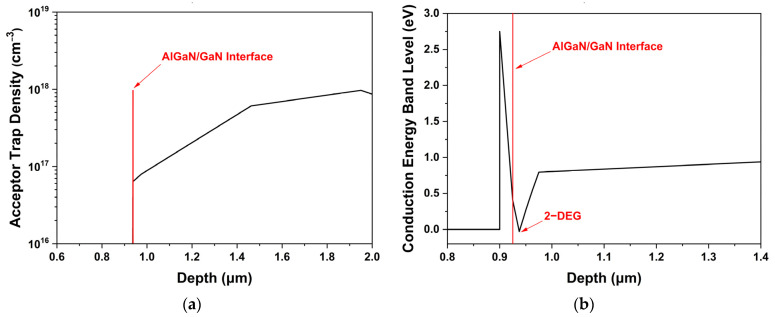
(**a**) Acceptor trap doping profile for the GaN buffer layer as a function of depth; (**b**) conduction energy band level of the simulated device as a function of depth.

For the simulation study, it is important to apply the appropriate electrical and thermal parameters for each material and simulation model to ensure data reliability and consistency with actual device operating characteristics. Figure 3 shows the acceptor trap density and conduction energy band of the AlGaN/GaN interface. Acceptor trap doping by Fe (Iron) was exploited in the GaN buffer layer to prevent the electron punch-through effect and minimize the substrate leakage current to improve $V_{\mathrm{BD}}$ [18]. As shown in Figure 3a, we used the Gaussian acceptor doping profile to consider diffusion as actual doping, in which the doping concentration gradually decreased with the peak trap concentration of 10^18^/cm^3^; thus, the acceptor doping concentration at the AlGaN/GaN interface was set to 6.376 × 10^16^/cm^3^ [19].

Figure 3b shows the conduction energy band level of the simulated device as a function of depth. When a heterojunction between AlGaN and GaN is formed, the conduction band and valence band throughout the material must bend to form a continuous Fermi level. The conduction band offset of AlGaN and GaN layer transfers electrons from AlGaN to GaN layer, from states with higher to lower energy level using band bending. The electrons that are transferred to GaN layer are confined to a small region in the channel layer near the hetero interface, which is called the 2-DEG, as shown in Figure 3b. It was confirmed that a 2-DEG potential well was formed at a depth of 0.0125 μm below the AlGaN/GaN interface with a 2-DEG charge carrier density of 4.35 × 10^12^/cm^2^. All the electrical and thermal parameters of AlGaN and GaN used for the simulation are summarized in Table 2 [20,21].

The heat generated in the device due to the self-heating effect (SHE) causes phonon scattering, which reduces electron mobility and degrades device performance. Therefore, it is essential to consider the SHE for an accurate simulation of the HEMT operational characteristics [22,23]. To include the SHE for AlGaN/GaN HEMTs in the simulation, we applied the lattice heat flow equation as:

(1) $$C\frac{\partial T_{L}}{\partial t}=\nabla \left(\kappa \nabla T_{L}\right)+H$$

where C is the heat capacitance per unit volume, κ is the thermal conductivity, $T_{L}$ is the local lattice temperature, and H is a heat generation term [24,25,26]. When the carrier transport is handled by the drift-diffusion approximation, H in Equation (1) has the simplified form:

(2) $$H=\left(\vec{J_{n}}+\vec{J_{p}}\right)\cdot \vec{E}$$

where $\;\vec{J_{n}}$ and $\vec{J_{p}}$ are the electron and hole current densities, respectively, and  E → is the electric field [27].

The thermal conductivity model which is vital for the calculation of the SHE in the simulation can be expressed as:

(3) $$\kappa \left(T\right)=\left(\mathrm{TC}.\mathrm{CONST}\right)/{\left(T_{L}/300\right)}^{\mathrm{TC}.\mathrm{NPOW}}$$

where TC.CONST is a thermal conductivity constant for each material at 300 K, and TC.NPOW is a thermal conductivity factor which is an experimental value for each material in the thermal conductivity model. With Equation (3), the thermal conductivity of a material according to the lattice temperature can be calculated by setting the appropriate TC.CONST and TC.NPOW parameters. The applied thermal conductivity constants of GaN and AlGaN are also shown in Table 2 [28,29].

**Figure 4:**
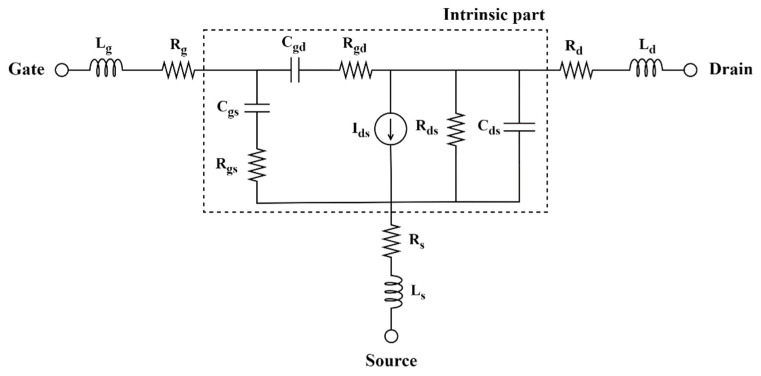
Small-signal equivalent circuit of HEMT.

Because RF characteristics can deteriorate with the application of the FP, we analyzed the frequency characteristics according to the parasitic capacitances. Figure 4 shows the small-signal equivalent circuit of HEMTs which can be divided into intrinsic part (emphasized by a dashed frame) and extrinsic part. The extracted values for all extrinsic and intrinsic small-signal parameters of simulated basic T-gate structure are listed in Table 3 [30]. Current gain and unilateral power gain were used to determine the $f_{T}$ and the maximum oscillation frequency ($f_{\max}$), respectively. Equations (4) and (5) explain the $f_{T}$ and $f_{\max}$, according to Figure 4:

(4) $$f_{T}=\frac{g_{m}}{2\pi \left(C_{\mathrm{gs}}+{\;C}_{\mathrm{gd}}\right)}\approx \frac{g_{m}}{2{\pi C}_{\mathrm{gs}}}$$

(5) $$f_{\max}=\frac{f_{T}}{2\sqrt{{\pi f}_{T}C_{\mathrm{gd}}\left(R_{s}+R_{g}+R_{\mathrm{gs}}+2{\pi L}_{s}\right)+G_{\mathrm{ds}}\;\left(R_{s}+R_{g}+R_{\mathrm{gs}}+{\pi f}_{T}L_{s}\right)}}\approx \sqrt{\frac{f_{T}}{8{\pi R}_{g}C_{\mathrm{gd}}}}$$

where $C_{\mathrm{gs}}$ and $C_{\mathrm{gd}}$ represent the gate-to-source capacitance and gate-to-drain capacitance, respectively. These parasitic capacitances dominate the decrease in $f_{T}$ according to Equation (4); thus, $C_{\mathrm{gs}}$ and $C_{\mathrm{gd}}$ must be reduced to obtain higher $f_{T}$. The $R_{s}$, $R_{g}$, $R_{\mathrm{gs}}$, and $G_{\mathrm{ds}}$ are the source resistance, gate resistance, gate-to-source resistance, and output conductance, respectively [31]. As denoted in Equation (5), decreasing the denominator components, such as $R_{g}$ and $C_{\mathrm{gd}}$, increases the $f_{\max}$.

In addition, the Fermi–Dirac distribution, Auger recombination, Shockley–Read–Hall recombination, and polarization models were applied to the simulation to calculate the basic operating characteristics of the device. The Selberherr model was used to calculate the temperature dependent impact ionization phenomenon caused by the electric field in the channel layer concentrated under the gate electrode during device operation [32].

## 3. Results

### 3.1. Matching of Simulated Data with Measured Data of a Basic T-Gate HEMT

**Figure 5:**
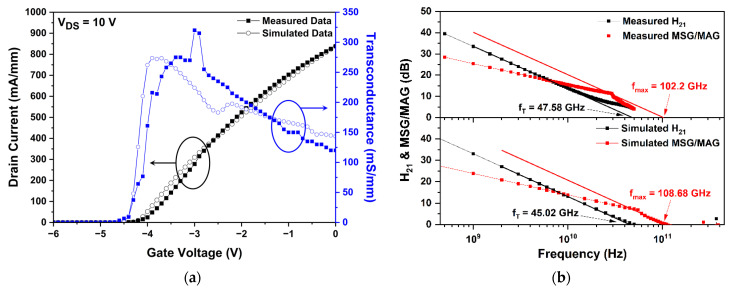
(**a**) Overlay of measured and simulated drain current-gate voltage ($I_{\mathrm{DS}}$-$V_{\mathrm{GS}}$) transfer characteristics of a basic T-gate HEMT; (**b**) measured and simulated frequency characteristics of a basic T-gate HEMT at drain voltage ($V_{\mathrm{DS}})$ = 20 V, gate voltage ($V_{\mathrm{GS}})$ = −3.2 V.

To confirm the reliability of the simulation, the drain current-gate voltage ($I_{\mathrm{DS}}$-$V_{\mathrm{GS}}$) transfer and small-signal characteristics between the simulated and measured data of the fabricated basic T-gate AlGaN/GaN HEMT were matched. Figure 5a shows the overlay of the measured and simulated values for the $I_{\mathrm{DS}}$-$V_{\mathrm{GS}}$ transfer characteristics of a basic T-gate HEMT at a drain voltage of 10 V. The simulated threshold voltage ($V_{\mathrm{th}}$) was −4.4 V, which matches well with the measured value of −4.5 V. The measured and simulated values of the maximum transconductance ($G_{m}$) were 275.00 mS/mm and 273.68 mS/mm, respectively, at a drain bias of 10 V. In addition, the drain current at a gate voltage of 0 V ($I_{\mathrm{dss}}$) was 837.65 mA/mm in the measured data and 838.24 mA/mm in the simulated data, both of which are similar. A dip of the simulated drain current around gate voltage of −2.5 V was found since two different electron mobility models were used in simulation as described in Table 2 and it is not known exactly which criterion divides the low field and the high field. There could be a mismatch in drain current at the turning point of these two mobility models. Consequently, the reliability of the simulation study was confirmed by matching the simulated data with the measured data, the maximum error rate of which was only 2.2%. The DC measurements of the fabricated device were conducted using a HP4142B Modular DC Source/Monitor probe station and a Cascade Microtech Summit 12000 probe station.

The measured and simulated frequency characteristics of the basic T-gate structure were shown together in Figure 5b. The $f_{T}$ was defined at the point where the −20 dB/decade slope extension line of the current gain ($H_{21}$) becomes 0 dB. The point where the extension line of the −20 dB/decade slope at the intersection point of the maximum stable/available gain (MSG/MAG) becomes 0 dB was defined as $f_{\max}$ [33]. The bias voltage applied for the simulation of RF characteristics is $V_{\mathrm{DS}}$ = 20 V, $V_{\mathrm{GS}}$ = −3.2 V, where $f_{T}$ and $f_{\max}$ are the highest among the measured values of a basic T-gate structure. Simulated $f_{T}$ and $f_{\max}$ were 45.02 GHz and 108.68 GHz, and measured $f_{T}$ and $f_{\max}$ were 47.58 GHz and 102.2 GHz, respectively, for the basic T-gate structure. The simulated $f_{T}$ and $f_{\max}$ values were well matched to measured values of those with the error rate within 6.3%. The small-signal RF performance of the fabricated device was measured from 0.5 GHz to 50 GHz with a PNA-X N5245A network analyzer.

### 3.2. Comparative Analysis of a Basic T-Gate and Slant A, Slant B, and Slant C Structures

**Figure 6:**
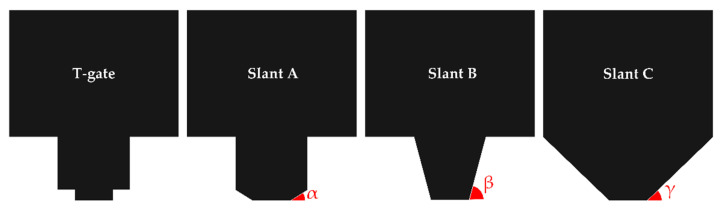
Schematics of various gate electrode structures for the AlGaN/GaN HEMT: A basic T-gate structure, slant A structure, slant B structure, and slant C structure.

To increase the $V_{\mathrm{BD}}$, three different slant-gate structures were suggested, as shown in Figure 6. Each slant-gate structure was slantly connected to the gate electrode edge point where the 1st and 2nd SiN passivation layers meet, respectively. Figure 6 shows a standard basic T-gate, slant A, slant B, and slant C structures with maintaining an $L_{\mathrm{Gate}-\mathrm{Foot}}$ of 0.18 μm as in Table 1. The slope of the slanted gate was 32° for slant A (α), 75° for slant B (β), and 44° for slant C (γ). The lengths of the slant region of slant A, slant B, and slant C are 0.26 μm, 0.31 μm, and 0.43 μm, respectively.

#### 3.2.1. Simulation of DC Characteristics

**Figure 7:**
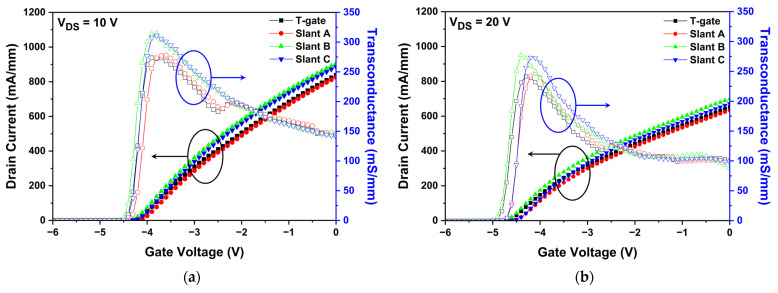

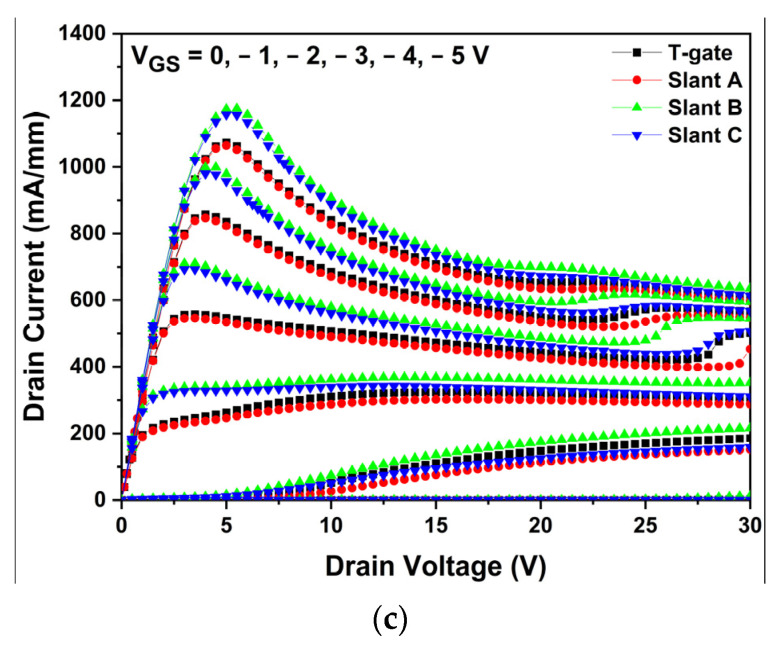
Simulated results of the DC characteristics of the slant-gate structures: $I_{\mathrm{DS}}$-$V_{\mathrm{GS}}$ transfer characteristics at (**a**) $V_{\mathrm{DS}}$ = 10 V and (**b**) $V_{\mathrm{DS}}$ = 20 V; and (**c**) drain current–drain voltage ($I_{\mathrm{DS}}$-$V_{\mathrm{DS}}$) output characteristics at gate voltages of −5, −4, −3, −2, −1, and 0 V.

We first simulated the DC characteristics of the slant-gate structure HEMTs and compared them to those of the basic T-gate HEMT. As shown in Figure 7a,b, the $I_{\mathrm{DS}}$-$V_{\mathrm{GS}}$ transfer characteristics, such as the threshold voltage, drain current, and transconductance, at a drain voltage of 10 V and 20 V were compared. In Figure 7a, it can be observed that the slant gate structures except slant A improve both the drain current and transconductance than basic T-gate structure, with constant $V_{\mathrm{th}}$. In Figure 7b, the SHE becomes severe at the drain voltage of 20 V, which shows more pronounced drain current reduction and transconductance decrease than those at the drain voltage of 10 V. With the increase in drain voltage, more heat is generated, which decreases the mobility of the electrons due to phonon scattering. By comparing Figure 7a,b, the thermal operational degradation confirms that a higher electric field between the drain and source results in less drain current because of the severe SHE. Figure 7c shows the drain current–drain voltage ($I_{\mathrm{DS}}$-$V_{\mathrm{DS}}$) output characteristics and decrease in drain current for all structures is observed as the drain voltage increases due to the SHE. When the higher drain voltage was applied, higher electric fields generated more heat, resulting in phonon scattering that reduced electron mobility and drain current density [34]. Moreover, you can also find the slight increase in the drain current at a certain high drain voltage, which is called the kink effect. The kink effect is caused by the carrier trapping process, occurred by the hot electrons which contribute to the trap formation in the AlGaN barrier or GaN buffer layer. The hot electrons in the 2-DEG channel generated under high field acceleration of high drain bias under gate electrode with sufficient energy could be injected into the adjacent AlGaN barrier or GaN buffer layer, where they can be captured by donor-like traps [35,36,37]. In addition, the proposed slant C structure reduces the device on-resistance ($R_{\mathrm{ON}}$) by 9%, compared to the basic T-gate structure [38,39,40].

**Figure 8:**
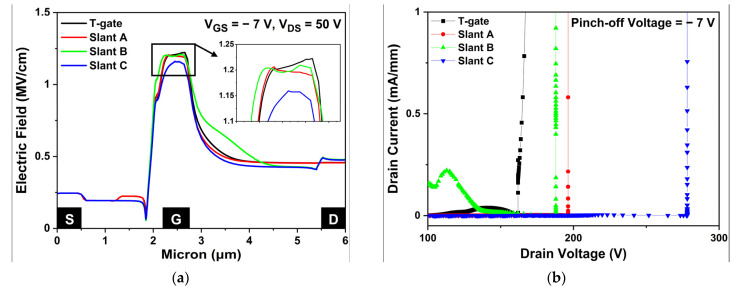
(**a**) Electric field across the two-dimensional electron gas channel layer between source and drain at $V_{\mathrm{GS}}$ = −7 V, $V_{\mathrm{DS}}$ = 50 V; (**b**) breakdown characteristics at a pinch-off of $V_{\mathrm{GS}}$ = −7 V.

Figure 8a shows the electric field in the 2-DEG channel layer which shows a reduced peak electric field in slant C structure by 5%, compared to the basic T-gate structure. In Figure 8b, the three different slant gate structures demonstrated an increased $V_{\mathrm{BD}}$ compared with the basic T-gate structure. Since impact ionization cause a sufficient increase in the drain current due to the generation of electron-hole pairs in the channel region from a high electric field close to the gate, dispersing the electric field is effective in improving the breakdown voltage. The $V_{\mathrm{BD}}$ was extracted at the point where the drain leakage current exceeds 1 mA/mm, when the gate pinch-off voltage of −7 V was applied to ensure the off-state of the device. Those were 167.44, 196.32, 187.93, and 278.13 V for the T-gate, slant A, slant B, and slant C structures, respectively. The $V_{\mathrm{BD}}$ of the slant C structure increased the most to 278.13 V, which is 66% higher than the $V_{\mathrm{BD}}$ of 167.44 V of the basic T-gate structure.

#### 3.2.2. Simulation of RF Characteristics

**Figure 9:**
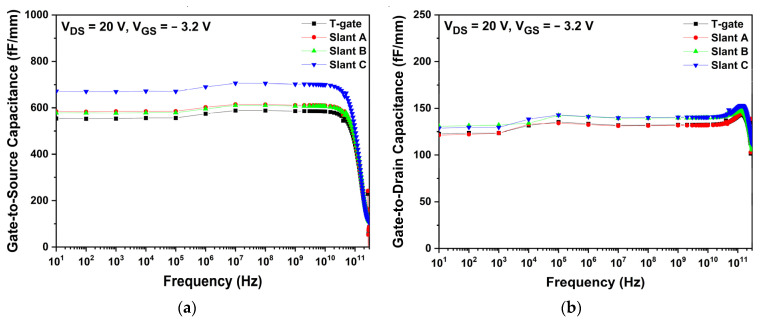
Capacitance characteristics as a function of frequency for different gate structures: (**a**) gate-to-source capacitance; (**b**) gate-to-drain capacitance.

In Figure 9, the $C_{\mathrm{gs}}$ and $C_{\mathrm{gd}}$ of each gate structure are compared at a drain voltage of 20 V and a gate voltage of −3.2 V. The structural change in the gate electrode affected both $C_{\mathrm{gs}}$ and $C_{\mathrm{gd}}$. Because $L_{\mathrm{gs}}$ were shorter than $L_{\mathrm{gd}}$, as described in Table 2, $C_{\mathrm{gs}}$ were generally larger than $C_{\mathrm{gd}}$. As shown in Figure 9a, the slant C structure showed the largest $C_{\mathrm{gs}}$ because the distance between the drain and gate electrode is relatively shorter. The $C_{\mathrm{gd}}$ of the T-gate, slant A, slant B, and slant C structures were almost the same at approximately 130 fF/mm, as shown in Figure 9b. Compared with $C_{\mathrm{gs}}$, only a small change in $C_{\mathrm{gd}}$ was observed for various gate structures. For the same reason that $L_{\mathrm{gd}}$ was longer than $L_{\mathrm{gs}}$, $C_{\mathrm{gd}}$ was less affected by the changes in the gate structure [41].

**Figure 10:**
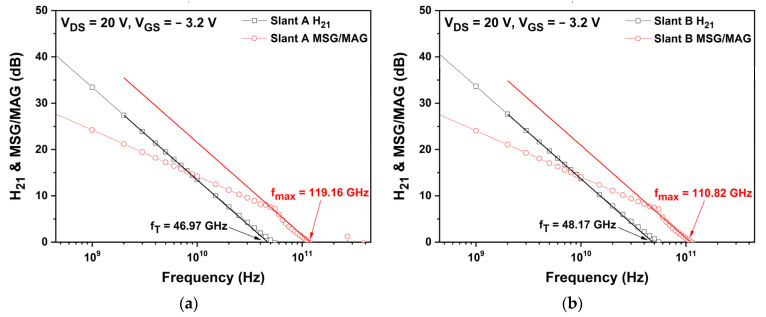

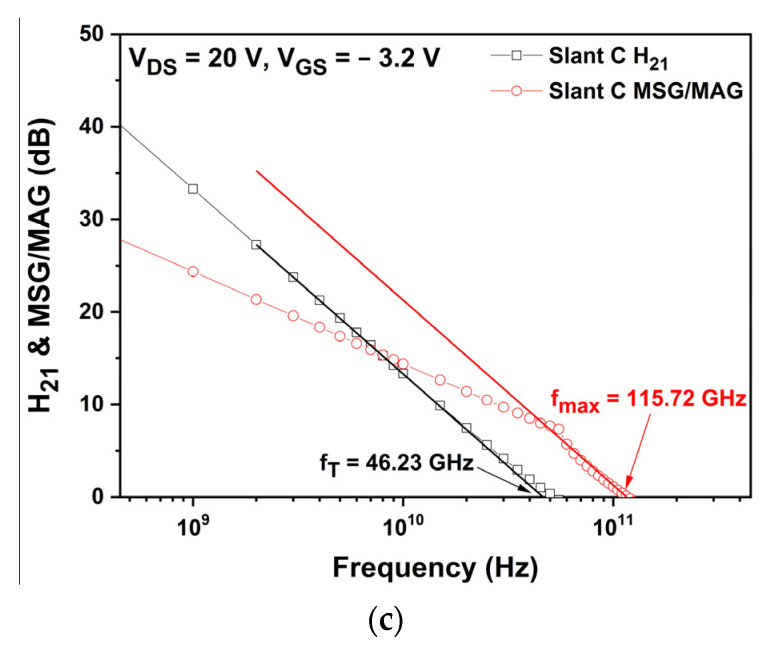
Cut-off frequency ($f_{T}$) and maximum frequency ($f_{\max}$) of the slant-gate structures at $V_{\mathrm{DS}}$ = 20 V, $V_{\mathrm{GS}}$ = −3.2 V: (**a**) $H_{21}$ and MSG/MAG versus frequency for the slant A structure; (**b**) $H_{21}$ and MSG/MAG versus frequency for the slant B structure; and (**c**) $H_{21}$ and MSG/MAG versus frequency for the slant C structure.

Figure 10 shows the simulated $f_{T}$ and $f_{\max}$ of three slant-gate structures. The simulated $f_{T}$ values of the slant A, slant B, and slant C structures at a drain voltage of 20 V and a gate voltage of −3.2 V were 46.97, 48.17, and 46.23 GHz, respectively. According to Equation (4), the $f_{T}$ of the slant-gate structures may have been influenced by the $g_{m}$ and $C_{\mathrm{gs}}$. The $f_{\max}$ values of the slant A, slant B, and slant C structures were 119.16, 110.82, and 115.72 GHz, respectively. The $f_{\max}$ of the slant-gate structures increased due to the increase in $f_{T}$ and the decrease in $R_{g}$ according to Equation (5). When the slant-gate structures are applied, the $f_{T}$ is increased by up to 7% and the $f_{\max}$ is increased by up to 10%, compared to those for the basic T-gate structure, respectively. Through these results, it was confirmed that there was no degradation in the RF characteristics when the slant-gate structures were applied.

### 3.3. Comparative Analysis of the Operating Characteristics for the Slant C Structure with an Extended FP

**Figure 11:**
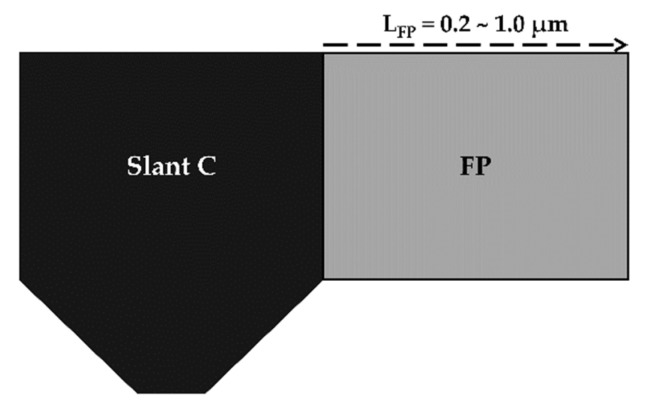
Schematic of the slant C with a drain-side extended field-plate (FP) applied gate structure.

Since the slant C structure demonstrated the highest $V_{\mathrm{BD}}$ among the slant-gate structures, we applied a drain-side extended FP to the slant C structure and further improved the $V_{\mathrm{BD}}$. Figure 11 shows the schematic of the slant C with a drain-side extended FP applied gate structure. Except for the FP length, which was increased from 0.2 μm to 1.0 μm, all the remaining structural variables of the device were fixed. We analyzed five different FP lengths, i.e., 0.2, 0.4, 0.6, 0.8, and 1.0 μm, to determine the optimum FP length.

#### 3.3.1. Simulation of DC Characteristics

**Figure 12:**
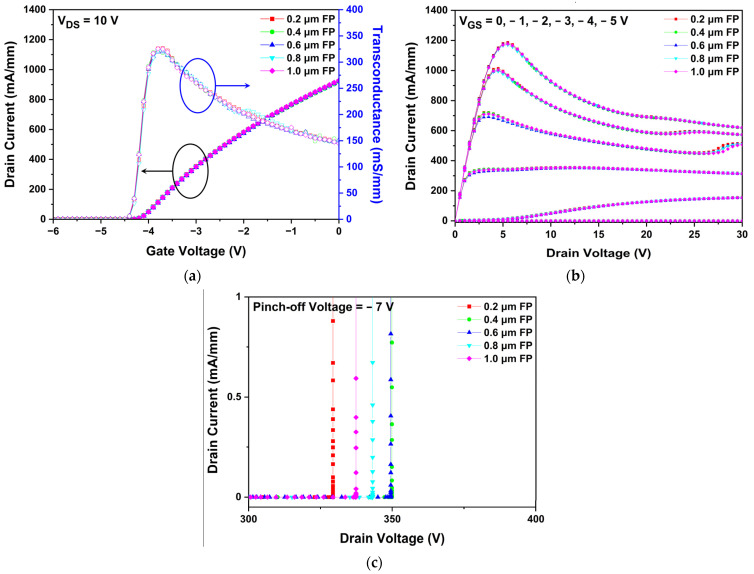
Simulated results of the DC characteristics of slant C structure with various FP lengths: (**a**) $I_{\mathrm{DS}}$-$V_{\mathrm{GS}}$ transfer characteristics at $V_{\mathrm{DS}}$ = 10 V; (**b**) $I_{\mathrm{DS}}$-$V_{\mathrm{DS}}$ transfer characteristics at gate voltages of −5, −4, −3, −2, −1, and 0 V; and (**c**) breakdown characteristics at a pinch-off of $V_{\mathrm{GS}}$ = −7 V.

To determine the optimum FP length, the DC characteristics of the device, such as $I_{\mathrm{DS}}$-$V_{\mathrm{GS}}$ transfer, $I_{\mathrm{DS}}$-$V_{\mathrm{DS}}$ output, and the breakdown voltage characteristics were simulated and compared by increasing the FP length up to 1.0 μm. The $V_{\mathrm{th}}$, $G_{m}$, and $I_{\mathrm{dss}}$ did not change significantly with the extension of FP as shown in Figure 12a. As shown in Figure 12b, the thermal operational degradation due to the SHE was detected in the $I_{\mathrm{DS}}$-$V_{\mathrm{DS}}$ output characteristics, but there were no significant differences in the overall curve trends and $R_{\mathrm{ON}}$ according to the FP length. Similarly, a kink effect as shown in Figure 7c was also found near a drain voltage of 20 V or 25 V.

Figure 12c presents the $V_{\mathrm{BD}}$, showing the drain current as a function of drain voltage for different FP lengths. The $V_{\mathrm{BD}}$ is strongly related to the FP length because the extension of the gate edge point redistributes the peak electric field in the channel layer. All breakdown voltages were much larger than that of the slant C structure without the FP. The slant C structure with an extended FP length of 0.4 μm showed the maximum $V_{\mathrm{BD}}$ of 349.87 V, which is an increase of 26% compared with that of the slant C structure without the FP. A further increase in the FP length beyond 0.6 μm decreases the $V_{\mathrm{BD}}$ because the electric field that increases as it approaches the drain electrode has more influence than the electric field dispersed by the FP. From the above results, it can be observed that when FP was applied to the slant C structure, other DC characteristics were almost maintained, but only the $V_{\mathrm{BD}}$ was affected.

#### 3.3.2. Simulation of RF Characteristics

**Figure 13:**
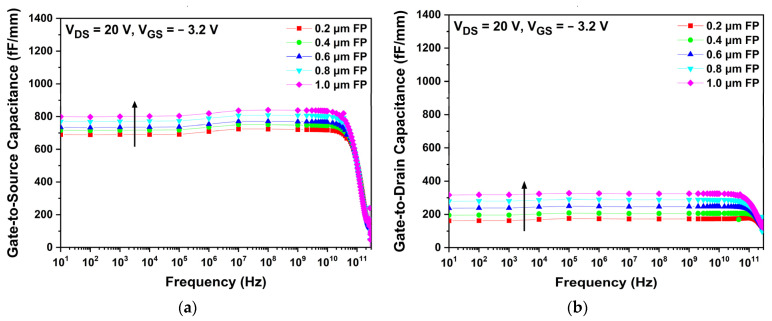
Capacitance characteristic variations for different lengths of drain-side extended FP: (**a**) gate-to-source capacitance; (**b**) gate-to-drain capacitance.

As expected, the sum of the parasitic capacitances increased with extending FP length, which affects the operational frequencies of the AlGaN/GaN HEMT. Figure 13 shows the capacitance variations for different FP lengths. Since $L_{\mathrm{gs}}$ is shorter than $L_{\mathrm{gd}}$, $C_{\mathrm{gs}}$ shows generally larger values than $C_{\mathrm{gd}}$ due to the fact that the capacitance is inversely proportional to the distance between the electrodes. Both $C_{\mathrm{gs}}$ and $C_{\mathrm{gd}}$ tend to increase with respect to FP length as shown in Figure 13a,b. Drain-side extended FP not only affects $C_{\mathrm{gd}}$ but also $C_{\mathrm{gs}}$. The reason is that in addition to the FP’s own extrinsic capacitance, there is also intrinsic capacitance beneath the gate edges (gate-to-source and gate-to-drain) due to the depletion region. The FP regulates the depletion region by uniform distribution of the electric field beneath both gate edges. The reduction in the electric field results in suppression and extension of the channel depletion region, hence raising the capacitance [42].

**Figure 14:**
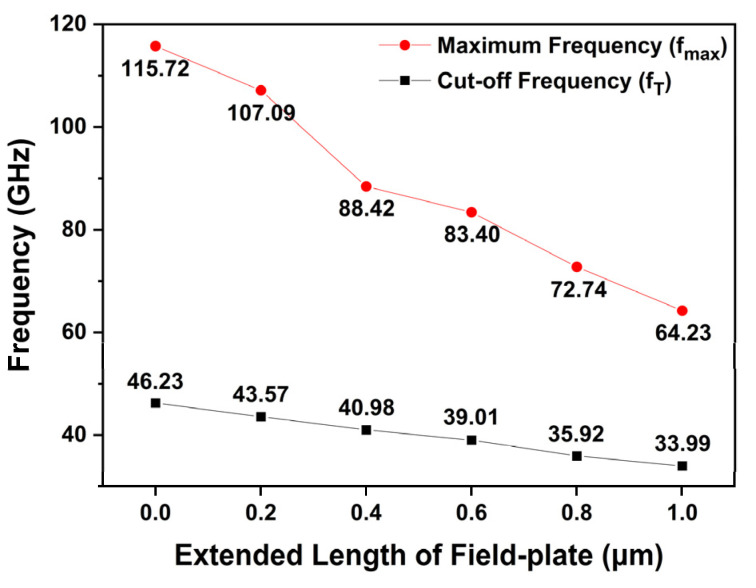
Simulated $f_{T}$ and $f_{\max}$ as a function of FP length at $V_{\mathrm{DS}}$ = 20 V and $V_{\mathrm{GS}}$ = −3.2 V.

Figure 14 shows the simulated $f_{T}$ and $f_{\max}$ values for different FP lengths at the drain voltage of 20 V and gate voltage of −3.2 V. The $f_{T}$ and $f_{\max}$ of the slant C structure, without any FP, were 46.23 GHz and 115.72 GHz, respectively. The $f_{T}$ was simulated to be 43.57, 40.98, 39.01, 35.92, and 33.99 GHz for the slant C structure with FP lengths of 0.2, 0.4, 0.6, 0.8, and 1.0 μm, respectively. The $f_{T}$ showed a tendency to decrease by approximately 5–9% as the FP length was extended by 0.2-μm-steps. As $C_{\mathrm{gs}}$ and $C_{\mathrm{gd}}$ raise as FP length increases, $f_{T}$ decreases by Equation (4). The $f_{\max}$ also decreased as the FP length was extended. The $f_{\max}$ was simulated to be 107.09, 88.42, 83.40, 72.74, and 64.23 GHz for the slant C structure with FP lengths of 0.2, 0.4, 0.6, 0.8, and 1.0 μm, respectively. Comparing the $f_{\max}$ of the slant C structure based on the different FP lengths, it can be observed that the $f_{\max}$ decreases by 6% to 21% with the 0.2 μm step increase in FP. The $f_{\max}$ tends to decrease as $f_{T}$ decreases and $C_{\mathrm{gd}}$ increases according to Equation (5). This finding shows the dependence of a noticeable reduction in $f_{T}$ and $f_{\max}$ values with respect to FP length. Consequently, we can conclude that FP length of 0.4 µm is superior in performances with different lengths of FP, considering both the high breakdown voltage and high frequency characteristics simultaneously.

## 4. Discussion

In this paper, we conducted simulations of the DC and RF characteristics for various slant-gate-based structures and studied the trade-off between $V_{\mathrm{BD}}$ and $f_{T}$. Among the slant A, slant B, and slant C structures, the slant C structure demonstrated the highest $V_{\mathrm{BD}}$. It is meaningful that the breakdown voltage can be greatly improved without a significant change in the fabrication process of conventional T-gate structure. In addition, it was found that $f_{T}$ and $f_{\max}$ were enhanced together with the improvement of the $V_{\mathrm{BD}}$ by employing the slant-gate structures. Thereafter, with varying lengths of drain-side extended FP applied, the slant C structure with a FP length of 0.4 μm demonstrated the highest $V_{\mathrm{BD}}$. However, the increase in FP length was inevitably accompanied by the decrease in $f_{T}$ and $f_{\max}$ due to parasitic capacitances.

Table 4 presents a summary of the DC and RF characteristics for the optimum two different slant-gate-based structures of the AlGaN/GaN HEMT. Compared with the basic T-gate structure, it was shown that the $V_{\mathrm{BD}}$, $f_{T}$, and $f_{\max}$ are all increased when the slant C structure is applied. The $V_{\mathrm{BD}}$ of the slant C structure with 0.4-μm FP appears higher, but the $f_{T}$ and $f_{\max}$ are decreased. The slant C structure increases the $V_{\mathrm{BD}}$ by 66%, and the slant C structure with a 0.4 μm FP increases the $V_{\mathrm{BD}}$ by 109% compared with the basic T-gate structure. Another noteworthy advantage of the proposed slant-gate-based structures is that they increase the $V_{\mathrm{BD}}$ while maintaining the $V_{\mathrm{th}}$ of the HEMT.

## 5. Conclusions

In this study, we investigated the operational characteristics of AlGaN/GaN HEMTs with slant-gate-based structures using TCAD simulation. Unlike other structures that only improve the breakdown characteristic, the trade-off between the breakdown voltage and frequency characteristics of suggested slant-gate-based structures were analyzed. The simulation parameters were obtained from fabricated basic T-gate AlGaN/GaN HEMT devices to verify the reliability of the simulation results. We proposed two optimum slant-gate-based structures for AlGaN/GaN HEMTs with enhanced operational characteristics; the slant C structure can be an excellent choice to obtain both high breakdown voltage and high frequency characteristics. For high-power applications, the slant C structure with a 0.4-μm-long FP can greatly improve the breakdown voltage even if the frequency characteristics are degraded. The simulated results clearly show that the suggested slant-gate-based HEMTs are superior in performance over conventional T-gate HEMTs for future high-power and high-frequency applications.

## Figures and Tables

**Table 1:** Geometrical parameters of the basic T-gate structure and epitaxial layer used in the simulation.

Parameter	Value (μm)
① $L_{\mathrm{Gate}-\mathrm{Source}}$	1.05
② $L_{\mathrm{Gate}-\mathrm{Head}}$	0.8
③ $L_{\mathrm{Gate}-\mathrm{Middle}}$	0.34
④ $L_{\mathrm{Gate}-\mathrm{Foot}}$	0.18
⑤ $L_{\mathrm{Gate}-\mathrm{Height}}$	0.6
⑥ $L_{\mathrm{Gate}-\mathrm{Drain}}$	3.15
⑦ $L_{\mathrm{Sidewall}}$	0.2
1st passivation	0.05
2nd passivation	0.25
AlGaN barrier	0.025
GaN buffer	2
Nucleation layer	0.2

**Table 2:** Material parameters used for simulation at a temperature of 300 K (SRH: Shockley–Read–Hall).

Parameters	Units	GaN	AlGaN
Bandgap energy	eV	3.39	3.88
Electron affinity	eV	4.2	2.3
Relative permittivity	-	9.5	9.38
Low field electron mobility	cm^2^/V-s	1500	300
High field electron mobility	-	GANSAT Mobility Model	
Electron saturation velocity	cm/s	1.9 × 10^7^	1.12 × 10^7^
Hole saturation velocity	cm/s	1.9 × 10^7^	1.00 × 10^6^
Electron SRH lifetime	s	1.0 × 10^–8^	1.0 × 10^–8^
Hole SRH lifetime	s	1.0 × 10^–8^	1.0 × 10^–8^
TC.CONST	W/cm-K	1.3	0.4
TC.NPOW	-	0.43	0

**Table 3:** Extracted small-signal parameters for extrinsic elements at drain voltage ($V_{\mathrm{DS}})$ = 0 V and gate voltage ($V_{\mathrm{GS}})$ = 0 V and intrinsic elements at $V_{\mathrm{DS}}$ = 20 V and $V_{\mathrm{GS}}$ = −3.2 V.

Extrinsic Elements		Intrinsic Elements	
L_g_ = 2.58698 pH	R_g_ = 0.1299 Ω	C_gd_ = 13.9719 fF	R_gd_ = 266.914 Ω
L_d_ = 2.26741 pH	R_d_ = 16.082 Ω	C_gs_ = 58.5212 fF	R_gs_ = 0.00019 Ω
L_s_ = 2.05828 pH	R_s_ = 9.8563 Ω	C_ds_ = 4.22561 fF	R_ds_ = 725.569 Ω

**Table 4:** Summary of typical DC and RF characteristics for three different gate structures.

Parameters		Unit	Basic T-Gate	Slant C	Slant C with 0.4-μm FP
DC characteristics	Threshold voltage	V	−4.4	−4.4	−4.4
	Maximum transconductance	mS/mm	273.68	311.04	320.06
	$\mathrm{Drain}\;\mathrm{current}\;(@V_{\mathrm{GS}}$= 0 V)	mA/mm	838.24	885.98	920.00
	On-resistance	Ω-mm	3.06	2.78	2.79
	Breakdown voltage	V	167.44	278.13	349.88
RF characteristics	Cut-off frequency	GHz	45.02	46.22	40.98
	Maximum oscillation frequency	GHz	108.68	115.72	88.42
Johnson’s figure of merit		THz-V	7.54	12.86	14.34
